# Efficacy of an Electric Toothbrush With Monitor in Dental Plaque Removal: A Crossover Randomized Controlled Trial

**DOI:** 10.7759/cureus.55278

**Published:** 2024-02-29

**Authors:** Yasunori Yoshinaga, Akinori Oyama, Kimiko Ohgi, Naoki Maruo, Hiroaki Yamato, Nanako Tsuchimochi, Masanobu Nakagami, Ryuji Sakagami

**Affiliations:** 1 Section of Periodontology, Department of Odontology, Fukuoka Dental College, Fukuoka, JPN; 2 Oral Medicine Research Center, Fukuoka Dental College, Fukuoka, JPN; 3 Department of Research, HA-PPY Co. Ltd., Kumamoto, JPN

**Keywords:** oral hygiene, biofilm, image sensor, electric toothbrush, plaque-removal effect

## Abstract

Background and purpose: Plaque control is very important in the treatment of periodontitis. However, plaque is difficult to remove because one cannot see one's own oral cavity. The purpose of this study was to verify the plaque removal effect of a prototype device that has a built-in image sensor in the head of an electric toothbrush, enabling the user to brush while checking the condition of the tooth surface on a monitor in real time and to assess their sense of use.

Materials and methods: The subjects were 10 fifth-year students from the Graduate School of Dental Science, Fukuoka Dental College, Fukuoka, Japan. The subjects were divided into those who used electric toothbrushes while having the condition of the tooth surface checked with a monitor (monitor group) and those without a monitor (non-monitor group). O’Leary plaque control records before and after brushing and the brushing time were measured, and questionnaires were given to the subjects after brushing. Scaling and professional tooth cleaning were performed after completing the questionnaire. One week later, subjects were switched to the opposite group and had the same measurements and questionnaires. The Wilcoxon signed-rank test was used to compare both groups before and after the examination at a 5% significance level.

Results: The monitor group had significantly better plaque removal than the non-monitor group. In addition, the monitor group spent significantly more time brushing than the control group.

Conclusion: Brushing while monitoring oral conditions in real time using an electric toothbrush with a built-in image sensor showed that significantly better plaque removal can be achieved with a longer brushing time.

## Introduction

Periodontitis is an inflammatory disease caused by dental plaques that form on tooth surfaces. Periodontal treatment generally begins with initial periodontal treatment, followed by periodontal surgical treatment, restorative treatment, and supportive periodontal therapy. However, plaque control is an essential part of each phase in this series of treatments. Mechanical removal with a toothbrush is thought to be more effective than chemical cleaning for removing bacterial plaque from tooth surfaces [1-4]. Good plaque control through self-care is essential for successful periodontal treatment. However, since patients brush their teeth daily, it takes time to learn the technique, and it is difficult to achieve complete plaque control [5,6].

In Japan, manual toothbrushes are often used for brushing because they are available at reasonable prices. To improve mechanical plaque control using a toothbrush, various techniques have been devised, such as flocking of the toothbrush, shape, hardness, length of bristles, size of the head, and length and angle of the gripping handle [7,8]. However, since many patients cannot achieve adequate plaque control with manual toothbrushes, various electric toothbrushes have been developed in recent years to further improve plaque control [6,9,10]. Electric toothbrushes were first commercially introduced with a back-and-forth motion in the early 1960s [11]. Subsequently, rotating brushes were developed, and more recently, higher-frequency vibrating brushes have been developed [12,13]. There are many reports that electric toothbrushes are more effective than manual toothbrushes in removing plaque, suggesting their usefulness [14,15]. Recently, sonic toothbrushes that use sonic vibration and electric toothbrushes based primarily on rotation have been developed [16]. One report suggested that there was no significant difference between the two types of electric toothbrushes in terms of plaque removal, while another report showed that electric toothbrushes with rotary motion were more effective [17,18].

Furthermore, as it is difficult to check the condition of the oral cavity on its own, it is difficult to master the proper brushing method to remove plaque. Therefore, we thought that it would be easier to remove plaque if it was possible to check the condition of the oral cavity while brushing and made a prototype of a device with a small built-in image sensor at the head of the rotary electric brush, which made it possible to brush while checking the tooth surface in real time using the monitor. This study aimed to verify the usefulness of this device and evaluate the patient’s sense of use.

## Materials and methods

Ethical clearance and informed consent

The approval for the study (approval number 425) was obtained from the Ethics Committee of Fukuoka Gakuen, Fukuoka, Japan, and was performed in accordance with the ethical standards as laid down in the 1964 Declaration of Helsinki and its later amendments or comparable ethical standards. All participants signed a paper informed consent form after understanding the content. The study was registered in the UMIN Clinical Trials Registry with trial ID UMIN000050249.

Toothbrush used

**Figure 1:**
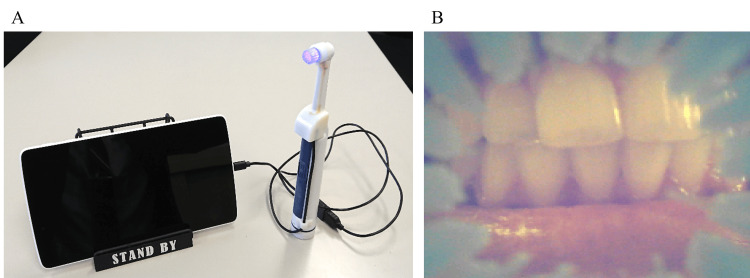
Prototype of the electric toothbrush in this study A. Electric toothbrush with a camera and display device. An image sensor is equipped in the head of the toothbrush. B. Display device image. Actual image displayed on the device monitor. Bristles of the brush are seen in the circumferential area.

An image sensor was built into the head of a rotating electric toothbrush (Figure 1). By connecting the image sensor to a tablet device, we were able to create a device that allows the subject to monitor the condition of the tooth surface in real time (HA-PPY Co., Ltd., Kumamoto, Japan).

Subjects and studied teeth

The subjects were 10 fifth-year students (seven men, three women) at the Graduate School of Dental Science, Fukuoka Dental College, Fukuoka, Japan; both men and women without age limitations were thought to have a certain level of knowledge and techniques about oral hygiene. A crossover study was conducted by classifying the subjects into two groups of five. All researchers were faculty members of the Graduate School of Dental Science; participants were briefed prior to the start of experiments that participating in the study would have no impact on their grades before asking for their consent to participate. The teeth studied were all teeth in the oral cavity of the subjects, except for wisdom teeth.

Study schedule and method

**Figure 2:**
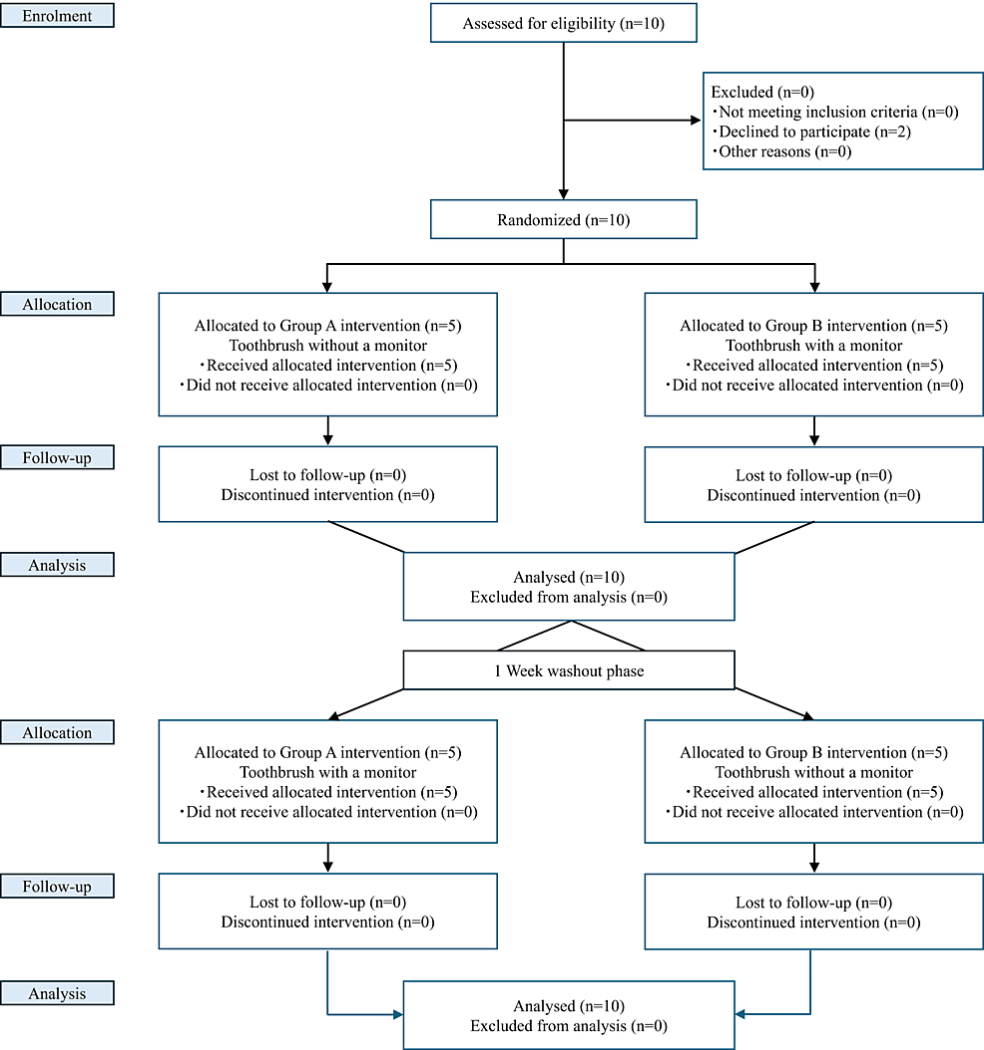
CONSORT flow and informed consent CONSORT: Consolidated Standards of Reporting Trials; n: number of participants

**Figure 3:**
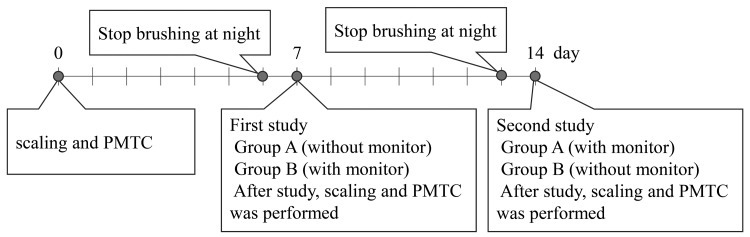
Experimental schedule PMTC: professional mechanical tooth cleaning

This study followed the Consolidated Standards of Reporting Trials (CONSORT) guidelines. The flow chart of the study is shown in Figure 2. The study protocol is outlined in Figure 3. Scaling and professional tooth cleaning were performed one week before the first test in all subjects. All subjects stopped brushing the night before the study, and the study itself was carried out during the afternoon.

For this randomized crossover trial, the participants were randomly assigned to Group A or B. In the first test, Group A performed brushing without a monitor, while Group B used a monitor. The O’Leary plaque control record (PCR) was measured before and after brushing before the subjects completed the questionnaires. Questionnaires were administered on the size of the toothbrush head, ease of holding it, and other aspects of its use. Brushing was terminated upon each subject arbitrarily feeling that the plaque was successfully removed, and the brushing time was measured for each subject. Finally, scaling and tooth surface cleaning were performed for all subjects.

In the second test conducted one week later, the subjects were switched to the opposite group to have the same measurements as the first test and completed the questionnaire.

The group that brushed without using a monitor was called the non-monitor group, whereas the group that brushed while checking with a monitor was called the monitor group.

Test of plaque adhesion

Plaque adherence was assessed using the O’Leary PCR [19]. Plaque was stained with a dental plaque staining solution (Prospec Plaque Disclosing Solution; GC Co., Tokyo, Japan), the tooth surface was divided into four (buccal, lingual, mesial, and distal), and the presence or absence of plaque adhesion on the cervical area of the teeth was evaluated. The adhesion rate was then measured. The removal rate was calculated using the following formula:

Removal rate = (Plaque adhesion rate before blushing - Plaque after brushing) / (Plaque adhesion rate before brushing)

Statistical analysis

The plaque adhesion rates before and after brushing, plaque removal rate, and brushing time between groups were compared using the crossover test, and the Wilcoxon signed-rank test was used for statistical analysis. Statistical analysis was performed using Statistical Product and Service Solutions (SPSS, version 25.0; IBM, Armonk, NY). Statistical significance was set at p<0.05.

## Results

Plaque adhesion level

**Figure 4:**
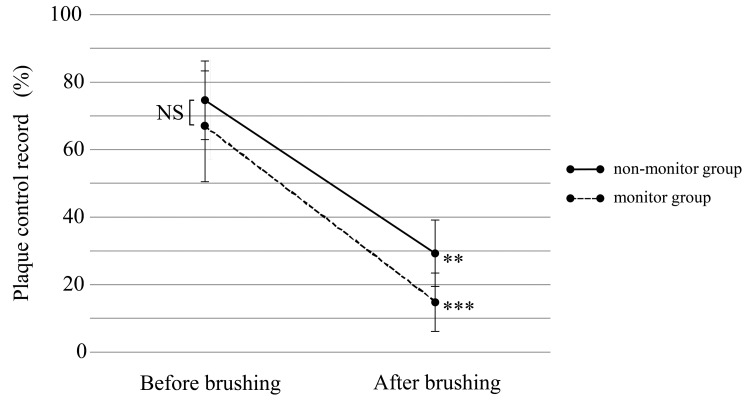
Plaque control record Plaque control record change between before and after brushing (including both groups A and B). Wilcoxon signed-rank test, vs. before brushing, ** p<0.01, *** p<0.001

PCR scores were measured and compared before and after brushing, and the difference in PCR scores between before and after brushing in each group was tested (Figure 4). The PCR scores before brushing were high in both groups, at 74.6±11.6% in the non-monitor group and 67.0±16.4% in the monitor group, with no significant difference (p=0.173). The PCR scores after brushing in the non-monitor group and monitor group were 29.3±9.8% and 14.8±8.6%, respectively, which meant that they had decreased significantly for both groups compared to before brushing (p=0.002 and p<0.001).

Plaque removal rate and brushing time

**Figure 5:**
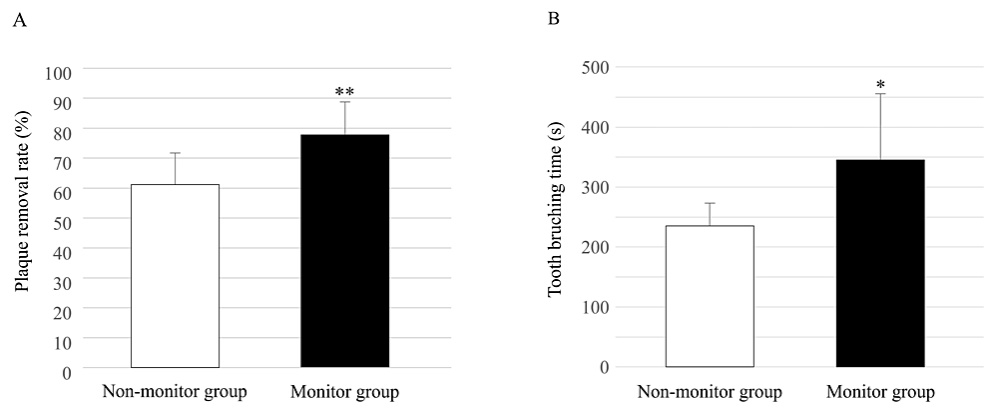
Plaque removal rate and tooth brushing time A. Plaque removal rate in groups with or without monitor. Wilcoxon signed-rank test, vs. the non-monitor group, ** p<0.01. B. Tooth brushing time in groups with or without monitor. Wilcoxon signed-rank test, vs. the non-monitor group, * p<0.05

The plaque removal rates were calculated and compared, taking the pre-brushing PCR score as 100 (Figure 5A). The plaque removal rate of brushing was significantly higher in the monitor group (77.8±11.1%) than in the non-monitor group (61.2±10.5%) (p=0.004). The brushing time was significantly longer in the monitor group compared to the non-monitor group (344.8±110.6 s vs. 235.2±38.1 s, respectively) (p=0.014) (Figure 5B).

Questionnaire results

**Figure 6:**
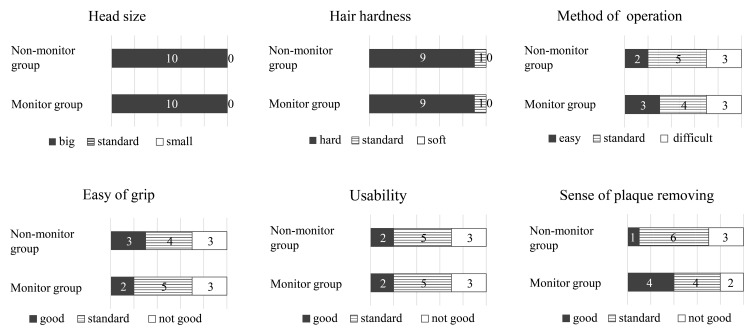
Results of the questionnaire

Despite using the same electric toothbrushes, the subjects were asked to complete a questionnaire on their sense of the use of the toothbrush when brushing without using a monitor and when brushing using the monitor and looking at the screen (Figure 6). There were no differences between the two groups in terms of “size of head,” “hardness of bristles,” and “sense of using while brushing.” The “ease of gripping the grip” was mentioned by one subject in the monitor group, changing from easy to grip to normal. In the monitor group, there was an increase in the number of subjects expressing that the “method of manipulating the electric toothbrush” was easy by one. With regards to “removal of plaques,” the response “removed well” increased by four subjects in the monitor group.

## Discussion

In this study, we investigated the plaque removal effect and sense of use of an electric toothbrush that can monitor the condition of the tooth surface in real time owing to the built-in image sensor in the head of the electric toothbrush. The electric toothbrush with a built-in image sensor used in this study was able to remove plaque more effectively than when used without a monitor. It is important for daily plaque control in periodontal treatment. Establishing high-level oral hygiene habits is not an easy task for patients themselves or the healthcare professionals who instruct them [5,6]. Difficulty in seeing one’s own oral cavity is considered a factor that affects self-care. By brushing while watching the monitor, the subjects were able to remove more plaque, demonstrating the effectiveness of the toothbrush in removing plaque.

In this study, we built a prototype of an electric toothbrush with a built-in image sensor showing the sensor data on a tablet screen so that the user can check the plaque in real time while brushing. At present, sonic toothbrushes are the mainstream electric toothbrushes, but there are reports that rotating electric toothbrushes are superior to sonic toothbrushes in plaque removal [20,21]. In addition, an image sensor was built into the toothbrush head, and a rotating electric toothbrush was used, considering that the image could be easily seen during brushing. An electric toothbrush has been compared to a manual toothbrush in a variety of study designs and settings [22]. Several studies have reported that an electric toothbrush performs better than a manual toothbrush in reducing plaque and gingivitis [10,23]. However, the cost of an electric toothbrush is substantially more than a manual toothbrush and comes with a variety of models and prices [15]. In addition, an electric toothbrush requires batteries to be recharged and replaced.

The 10 subjects were divided into two groups of five to undergo two tests in a crossover format such that each group of subjects would experience the use and non-use of the monitor. This method was used to equalize the brushing techniques of both groups, as we believed that individual differences in the brushing technique of the operator would have an impact on the results of plaque control. In addition, students at a dental school were included as study subjects to standardize the subjects’ knowledge level of toothbrushing methods and dentistry to a certain level. Many previous reports have set the brushing time to two to four minutes [24,25], but to reproduce clinical use, we allowed subjects to brush until they felt that they had brushed thoroughly, without limiting the brushing time. While van der Weijden et al. showed a close link between the brushing pressure and plaque-removal effect [26], we thought that standardizing the brushing pressure between subjects would be difficult as it varies depending on the toothbrush used, brushing method, and habits, among other factors [27]. We had subjects brush with the most comfortable brushing pressure without any particular provisions.

Even in the monitor non-use group, there was a significant reduction in plaque levels after brushing, with a relatively favorable mean PCR result of 23%. This demonstrates the effectiveness of the electric toothbrush used in this study for plaque removal. In addition, a comparison of the plaque removal rates between the two groups showed that the plaque removal rate was significantly higher in the monitor group than that in the monitor non-use group. We believe that a larger amount of plaque could be removed in the monitor group because the tooth surface could be monitored in real time. However, brushing time was significantly longer in the monitor group. We believe that, because subjects were able to monitor the tooth surface on the monitor, they were immersed in the brushing process, as if playing a game, which led to a longer brushing time. While a previous report suggested that two minutes of brushing is more effective than one minute of brushing for plaque removal [28], another report suggested that increasing the brushing time to three minutes or longer had no impact on plaque removal [29]. The findings from our experiments suggest that brushing teeth took more time when participants looked at the monitor, rather than resulting in an increase in brushing time, which would lead to an improvement in plaque removal rate.

There were no differences between groups in terms of “size of head,” “hardness of bristles,” and “sense of use while brushing.” We believe that these items did not vary because the electric toothbrush itself was the same between the two groups, with only the use or non-use of the monitor being the difference between the groups. However, the image sensor built into the toothbrush made the head of the toothbrush larger, and all subjects felt that the head was large. Furthermore, most subjects felt that the bristle was hard, and these points required improvements.

There were almost no differences between groups in terms of “method of manipulating the electric toothbrush,” “ease of gripping the grip,” and “sense of use,” but different subjects rated these differently. As operability and sense of use are important factors for ensuring continuous use of the device, it would be meaningful to add them as elements of analysis.

The number of subjects who responded that they had felt “plaque had been removed” increased with monitoring use. We believe that this is because the subject’s ability to independently monitor the brushing situation by themselves through the image on the monitor increased the feeling that the plaque was being removed. In addition, since a sense of accomplishment, such as the feeling that plaque has been removed, is an important factor in maintaining motivation for brushing, it is suggested that this sense is effective for getting the user to continue brushing at a high level.

The present study had several limitations. First, the sample size was small, with only 10 participants. The use of an electric toothbrush with a built-in image sensor should be investigated with more subjects in the future. In addition, there is a need for clinical trials to evaluate the effectiveness of toothbrushes with built-in imaging sensors for improving gingivitis and other oral health outcomes. Second, all participants were young dental students. The participants had good vision and no difficulty moving their arms. In the future, it will be necessary to consider other age groups, especially middle-aged and older adults. In addition, the fact that the participants were students with dental knowledge may have influenced the results of the questionnaire; future experiments should be conducted with participants who are not involved in dentistry.

## Conclusions

Our study showed that brushing teeth using an electric toothbrush with a built-in image sensor to monitor the tooth surface increased plaque removal. The brushing time increased because the subjects brushed their teeth more carefully while looking at the footage of their oral cavity. In addition, as patients themselves monitor their brushing habits, self-observation may lead to improved health awareness. In this study, we were able to evaluate the device's effectiveness in removing plaque and its usability. However, the effectiveness of the device on periodontal patients was not evaluated, and further studies are needed to evaluate its effectiveness in periodontal treatment.

## References

[1] Axelsson P, Lindhe J (1978). Effect of controlled oral hygiene procedures on caries and periodontal disease in adults. J Clin Periodontol.

[2] Axelsson P, Lindhe J (1981). Effect of controlled oral hygiene procedures on caries and periodontal disease in adults. Results after 6 years. J Clin Periodontol.

[3] Axelsson P, Lindhe J (1981). The significance of maintenance care in the treatment of periodontal disease. J Clin Periodontol.

[4] Axelsson P, Nyström B, Lindhe J (2004). The long-term effect of a plaque control program on tooth mortality, caries and periodontal disease in adults. Results after 30 years of maintenance. J Clin Periodontol.

[5] Jepsen S (1983). Role of manual toothbrushes in effective plaque control: Advantages and limitations. Proceedings of European Workshop on Mechanical Plaque Control. 1st Edition.

[6] Van der Weijden FA, Slot DE (2015). Efficacy of homecare regimens for mechanical plaque removal in managing gingivitis a meta review. J Clin Periodontol.

[7] Sharma NC, Qaqish J, Walters PA, Grender J, Biesbrock AR (2010). A clinical evaluation of the plaque removal efficacy of five manual toothbrushes. J Clin Dent.

[8] Stiller S, Bosma MLP, Shi X, Spirgel CM, Yankell SL (2010). Interproximal access efficacy of three manual toothbrushes with extended, x-angled or flat multitufted bristles. Int J Dent Hyg.

[9] Walmsley AD (1997). The electric toothbrush: a review. Br Dent J.

[10] Yaacob M, Worthington HV, Deacon SA, Deery C, Walmsley AD, Robinson PG, Glenny AM (2014). Powered versus manual toothbrushing for oral health. Cochrane Database Syst Rev.

[11] Chilton NW, Di-Do A, Rothner JT (1962). Comparison of the clinical effectivenes of an electric and a standard toothbrush in normal individuals. J Am Dent Assoc.

[12] Van der Weijden GA, Timmerman MF, Danser MM, Van der Velden U (1998). The role of electric toothbrushes: advantages and limitations. Proceedings of the European Workshop on Mechanical Plaque Control.

[13] Digel I, Kern I, Geenen EM, Akimbekov N (2020). Dental plaque removal by ultrasonic toothbrushes. Dent J (Basel).

[14] Elkerbout TA, Slot DE, Rosema NA, Van der Weijden GA (2020). How effective is a powered toothbrush as compared to a manual toothbrush? A systematic review and meta-analysis of single brushing exercises. Int J Dent Hyg.

[15] Thomassen TM, Van der Weijden FG, Slot DE (2022). The efficacy of powered toothbrushes: a systematic review and network meta-analysis. Int J Dent Hyg.

[16] Rosema N, Slot DE, van Palenstein Helderman WH, Wiggelinkhuizen L, Van der Weijden GA (2016). The efficacy of powered toothbrushes following a brushing exercise: a systematic review. Int J Dent Hyg.

[17] El-Chami H, Younis A, Brignardello-Petersen R (2021). Efficacy of oscillating rotating versus side-to-side powered toothbrushes on plaque and gingival index reduction: a systematic review. J Am Dent Assoc.

[18] Deacon SA, Glenny A-M, Deery C, Robinson PG, Heanue M, Walmsley AD, Shaw WC (2011). Different powered toothbrushes for plaque control and gingival health. Aust Dent J.

[19] O'Leary TJ, Drake RB, Naylor JE (1972). The plaque control record. J Periodontol.

[20] Klukowska M, Grender JM, Conde E, Goyal CR, Qaqish J (2014). A six-week clinical evaluation of the plaque and gingivitis efficacy of an oscillating-rotating power toothbrush with a novel brush head utilizing angled CrissCross bristles versus a sonic toothbrush. J Clin Dent.

[21] Klukowska M, Grender JM, Conde E, Ccahuana-Vasquez RA, Goyal CR (2014). A randomized 12-week clinical comparison of an oscillating-rotating toothbrush to a new sonic brush in the reduction of gingivitis and plaque. J Clin Dent.

[22] Klonowicz D, Czerwinska M, Sirvent A, Gatignol JP (2018). A new tooth brushing approach supported by an innovative hybrid toothbrush-compared reduction of dental plaque after a single use versus an oscillating-rotating powered toothbrush. BMC Oral Health.

[23] Kaklamanos EG, Kalfas S (2008). Meta-analysis on the effectiveness of powered toothbrushes for orthodontic patients. Am J Orthod Dentofacial Orthop.

[24] Saxer UP, Barbakow J, Yankell SL (1998). New studies on estimated and actual toothbrushing times and dentifrice use. J Clin Dent.

[25] Albertsson KW, van Dijken JW (2010). Awareness of toothbrushing and dentifrice habits in regularly dental care receiving adults. Swed Dent J.

[26] van der Weijden GA, Timmerman MF, Reijerse E, Snoek CM, van der Velden U (1996). Toothbrushing force in relation to plaque removal. J Clin Periodontol.

[27] Fraleigh CM, Mc Elhaney JH, Heiser RA (1967). Toothbrushing force study. J Dent Res.

[28] Slot DE, Wiggelinkhuizen L, Rosema NA, Van der Weijden GA (2012). The efficacy of manual toothbrushes following a brushing exercise: a systematic review. Int J Dent Hyg.

[29] Van der Weijden GA, Timmerman MF, Nijboer A, Lie MA, Van der Velden U (1993). A comparative study of electric toothbrushes for the effectiveness of plaque removal in relation to toothbrushing duration. Timerstudy. J Clin Periodontol.

